# An Empty Stomach or False Reassurance? Aspiration Pneumonitis Despite Prolonged Preoperative Fasting in a Low‐Risk Patient

**DOI:** 10.1002/ccr3.73296

**Published:** 2026-08-04

**Authors:** Tommaso Foggi Viligiardi, Giulia Borsotti, Gabriele Baldini, Chiara Adembri

**Affiliations:** ^1^ School of Anaesthesia and Intensive Care University of Florence Florence Italy; ^2^ Anaesthesia Unit for Head, Neck and Eye Surgery Careggi University Hospital Florence Italy; ^3^ Department of Health Sciences, Section of Anaesthesia and Intensive Care University of Florence Florence Italy

**Keywords:** airway management, aspiration pneumonitis, gastric fluid volume, gastric ultrasound, preoperative fasting, prolonged fasting

## Abstract

A healthy 38 year‐old male developed severe bronchospasm and aspiration pneumonitis during anesthesia induction despite 15‐h preoperative fasting. Imaging revealed bilateral pulmonary consolidations and gastric distension. This case visually highlights that prolonged fasting paradoxically fails to guarantee gastric emptiness, emphasizing the need for individualized risk assessment.

## Key Clinical Message

Prolonged preoperative fasting does not reliably predict gastric emptiness. Clinically significant residual gastric content may be present even in low‐risk patients, exposing them to aspiration risk despite adherence to standard fasting practices.

## 1 Introduction

Pulmonary aspiration of gastric contents during general anesthesia is an uncommon but potentially catastrophic complication, contributing significantly to anesthesia‐related morbidity and mortality [1]. International guidelines recommend minimum fasting intervals (2 h for clear liquids and 6 h for solids) to reduce this risk, while discouraging prolonged fasting [2, 3].

Despite these recommendations, excessive fasting remains widespread in clinical practice [4]. Importantly, emerging evidence indicates that fasting duration alone does not reliably predict gastric content, with clinically relevant residual volumes observed in a substantial proportion of elective surgical patients [5].

While specific clinical factors, such as the use of glucagon‐like peptide‐1 receptor agonists (GLP‐1 RAs), have recently renewed the debate on delayed gastric emptying [5], standard fasting intervals can fail even in healthy individuals due to high interindividual variability in gastric motility and physiological response to stress.

We report a case of aspiration pneumonitis occurring after prolonged fasting in a patient without identifiable risk factors, highlighting the limitations of a purely time‐based approach to aspiration risk stratification.

## 2 Case History

A 38‐year‐old male (height: 177 cm, weight: 72 kg, BMI: 23 kg/m^2^) was scheduled for elective septoplasty with bilateral turbinoplasty. His medical history included chronic allergic rhinitis and occasional smoking (3–4 cigarettes/day), with the last cigarette consumed more than 24 h prior to induction.

He denied gastrointestinal disease and was not taking medications known to delay gastric emptying. He reported fasting from both solids and liquids for more than 15 h. General anesthesia was induced with propofol 150 mg IV and remifentanil administered via target‐controlled infusion (effect‐site concentration titrated to 2.5 ng/mL).

Gentle bag‐mask ventilation was initiated without airway adjuncts, maintaining an Adjustable Pressure Limiting (APL) valve below 10 cmH_2_O to minimize the risk of gastric insufflation; the chest rose adequately with estimated tidal volumes of 400–500 mL and a respiratory rate of 10–12 breaths/min. Immediately thereafter, the patient developed severe hiccups followed by sudden hypoxemia (SpO_2_ 80%) and marked respiratory resistance. Rocuronium 0.6 mg/kg IV was promptly administered based on total body weight, and successful tracheal intubation was achieved on the first attempt (Cormack–Lehane grade I). Direct inspection revealed a small amount of clear fluid in the hypopharynx, initially interpreted as salivary secretions, without gross particulate matter or bile‐stained contents. Correct tube placement was confirmed by continuous end‐tidal CO_2_.

Promptly after intubation, mechanical ventilation was initiated in volume‐controlled mode with a protective tidal volume targeted at 6–8 mL/kg of predicted body weight (estimated at 500 mL). This immediately resulted in severely elevated peak airway pressures (39–41 cmH_2_O), reflecting critically low respiratory system compliance.

Auscultation revealed bilateral harsh breath sounds with diffuse expiratory wheezing. The capnogram waveform displayed a prolonged, upsloping phase III (‘shark‐fin’ pattern), highly characteristic of severe bronchospasm.

Consequently, despite confirmed tracheal intubation, compliance remained critically low and severe hypoxemia persisted, necessitating an immediate escalation of clinical care.

## 3 Differential Diagnosis, Investigations and Treatment

Given the abrupt onset of bronchospasm and severe hypoxemia immediately after induction in a previously low‐risk patient, the initial differential diagnosis included severe bronchospasm and a peri‐induction hypersensitivity reaction. Additional assistance was promptly requested.

On this basis, the patient was treated with dexamethasone 4 mg IV, methylprednisolone 40 mg IV, magnesium sulfate 2 g IV, inhaled salbutamol, and intravenous epinephrine (50 μg boluses, administered twice), reflecting concern for a possible evolving hypersensitivity reaction. Despite these interventions, respiratory mechanics remained impaired and gas exchange only partially improved.

Given the lack of a clear and sustained response to treatment, the initial diagnostic hypothesis was reconsidered, and alternative causes of acute respiratory deterioration were explored, including pulmonary aspiration.

**FIGURE 1:**
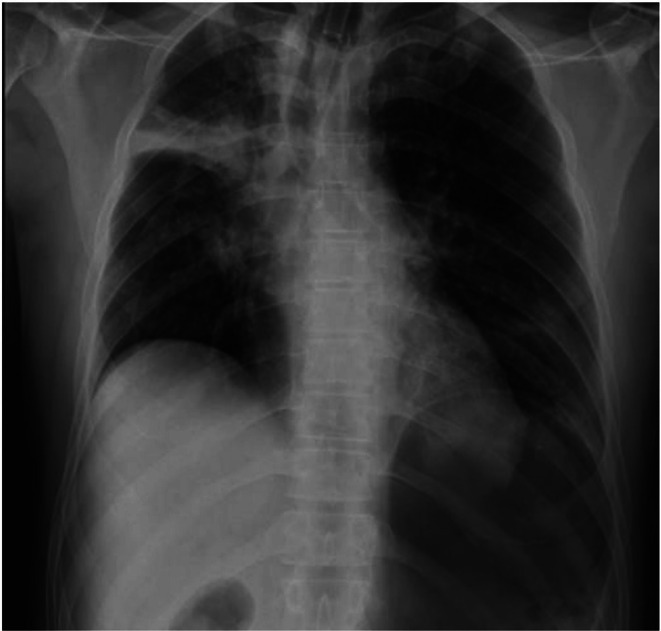
Chest X‐ray showing a right upper–middle lung field band‐like consolidation, compatible with upper lobe atelectasis. Endotracheal tube positioned approximately 5 cm from the carina, with mild rightward tracheal deviation. Marked distension of the gastric bubble is noted.

An urgent chest X‐ray revealed a right upper–middle lung field band‐like consolidation, compatible with upper lobe atelectasis, along with marked distension of the gastric bubble (Figure 1). Arterial blood gas analysis on FiO_2_ 100% showed severe hypoxemia (PaO_2_ 98 mmHg; P/F ratio 98) and hypercapnia (PaCO_2_ 58 mmHg).

**FIGURE 2:**
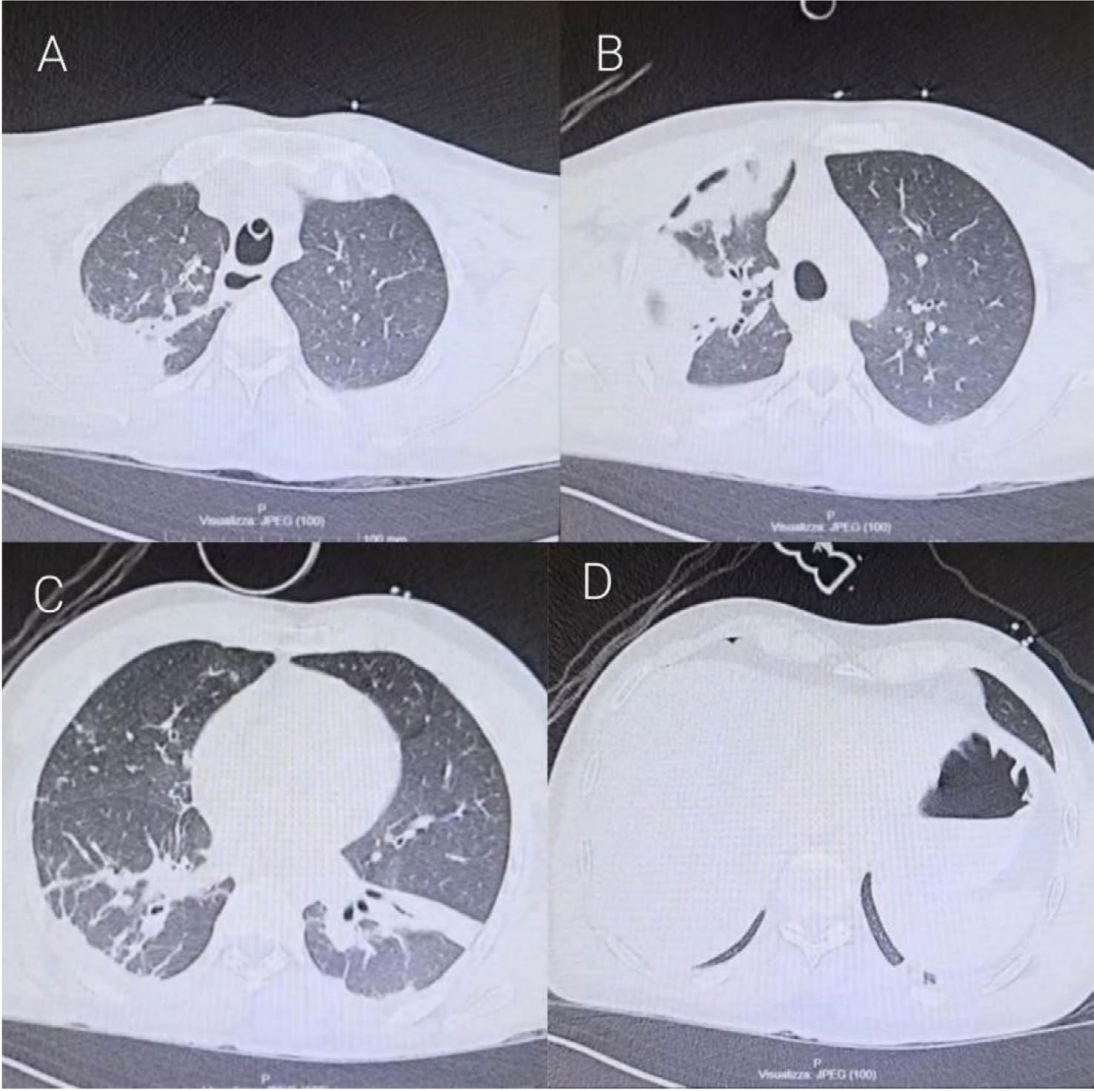
Series of chest CT scan slices demonstrating bronchioloalveolar distribution consolidations in the middle lobe. Additional consolidation areas with hypoventilatory appearance are visible: (A) in the right upper lobe, predominantly peripherally and along the fissures; (B) peripherally in the right lower lobe; (C) more limited, in the fissural and basal segments of the left upper lobe. (D) A more caudal chest CT slice showing a markedly distended gastric lumen with an air–fluid level at the fundus.

Pulmonary aspiration was strongly suspected, and the surgical procedure was consequently postponed. An urgent chest computed tomography (CT) was performed, which demonstrated a predominantly right‐sided distribution of consolidations specifically involving the right upper, middle, and lower lobes with more limited consolidative changes in the fissural and basal segments of the left upper lobe. The scan also confirmed marked gastric distension with a prominent air–fluid level at the fundus (Figure 2).

Flexible bronchoscopy revealed mild mucosal inflammation and scant secretions, without macroscopic evidence of particulate matter. The diagnosis of aspiration pneumonitis was initially clinical and radiographical, based on the classic presentation and imaging findings. This hypothesis was later supported by the negative results of the bronchoalveolar lavage (BAL) microbiological cultures, which ruled out pathogenic microorganisms and confirmed a sterile, chemical inflammatory process rather than an infectious pneumonia.

## 4 Conclusion and Results

The patient was transferred to the intensive care unit for management of acute respiratory failure. Hemodynamics remained stable without vasoactive support.

Respiratory function progressively improved. The patient was extubated approximately 16 h after the event and transferred to the ward after 24 h. He was discharged home 48 h after the episode without sequelae.

At follow‐up, the patient remained asymptomatic. Five weeks later, he underwent repeat septoplasty under general anesthesia without complications and was discharged home after 24 h of uneventful postoperative monitoring.

## 5 Discussion

This case highlights a clinically relevant discrepancy between prolonged fasting and actual gastric emptiness, resulting in pulmonary aspiration in a patient classified as low risk.

Pulmonary aspiration remains a significant contributor to anesthesia‐related morbidity and mortality, despite its relatively low incidence [1]. Current fasting guidelines are designed to reduce aspiration risk but rely on time‐based intervals rather than direct assessment of gastric content [2, 3]. However, gastric physiology is dynamic and influenced by multiple factors, including basal gastric secretion, interindividual variability in gastric motility, and perioperative stress responses. As a result, clinically significant residual gastric volumes may persist despite prolonged fasting, as demonstrated in recent observational data [4].

In our patient, the 15 h period of total fasting may have paradoxically contributed to the event. Classic and recent physiological data demonstrate that excessive restriction of oral fluid intake does not guarantee a reduced residual gastric volume; instead, it can paradoxically increase baseline gastric fluid volume and lower its pH due to unbuffered continuous gastric secretion [6].

Adherence to modern fasting guidelines, encouraging clear fluid intake up to 2 h preoperatively, might have stimulated gastric motility and promoted physiological emptying, reducing both volume and acidity.

In the present case, induction was conducted according to standard practice, with gentle bag‐mask ventilation and airway pressures maintained below thresholds typically associated with gastric insufflation. The occurrence of hiccups followed by acute respiratory deterioration suggests that transient physiological events during induction may have contributed to regurgitation, even in the absence of identifiable predisposing factors.

Alternative etiologies for the acute respiratory distress, such as mucus plugging or smoking‐related atelectasis, were thoroughly weighed. However, while atelectasis is a common cause of post‐induction hypoxemia, it rarely presents with sudden‐onset hiccups, intractable bronchospasm, and a ‘shark‐fin’ capnography pattern immediately following a smooth induction. While bag‐mask ventilation can theoretically cause gastric insufflation and subsequent fluid level on CT imaging, our strict adherence to airway pressures under 10 cmH_2_O makes iatrogenic gastric distension less likely as a primary cause. The clinical sequence of hiccups followed by immediate bronchospasm strongly supports a primary regurgitation event. Clinicians must remain aware that regurgitation is not always ‘witnessed’ or characterized by solid or bile‐stained material in the pharynx. Clear, non‐particulate gastric fluid can be easily misidentified as saliva or respiratory secretions in the acute phase [7].

From a diagnostic perspective, the initial hypothesis of a peri‐induction hypersensitivity reaction was clinically reasonable, given the abrupt onset of bronchospasm and hypoxemia in a previously low‐risk patient. This case illustrates the overlap in early clinical presentation between aspiration‐related bronchospasm and peri‐induction hypersensitivity reactions, which may lead to diagnostic uncertainty in the acute phase.

The subsequent lack of a sustained response to treatment prompted reconsideration of the initial diagnosis and expansion of the differential diagnosis. The identification of characteristic pulmonary infiltrates on imaging—predominantly distributed in the right lung but with bilateral involvement—together with marked gastric distension with an air–fluid level, strongly supported the clinical diagnosis of aspiration pneumonitis.

Importantly, the distinction between aspiration pneumonitis and aspiration pneumonia is clinically relevant. In this case, the initial clinical‐radiological suspicion was later confirmed by the absence of pathogenic microorganisms on bronchoalveolar lavage cultures. The rapid clinical improvement further supported a sterile chemical process rather than an infectious one, thereby justifying the decision to withhold antibiotic therapy [8].

Growing evidence indicates that residual gastric content is not uncommon even in appropriately fasted patients [4, 5]. In this context, point‐of‐care gastric ultrasound has emerged as a bedside tool capable of qualitatively and quantitatively assessing gastric content, with good diagnostic accuracy [9, 10]. In selected clinical scenarios, it may provide additional patient‐specific information and help identify unexpected aspiration risk.

These considerations are further reinforced by the increasing use of glucagon‐like peptide‐1 receptor agonists, which are associated with delayed gastric emptying and higher rates of residual gastric content despite adherence to fasting guidelines [5]. Although our patient was not receiving these agents, their widespread use in both diabetic and non‐diabetic populations highlights the limitations of relying exclusively on fasting duration for aspiration risk assessment.

## 6 Learning Points

- Fasting duration does not reliably correlate with gastric emptiness;
- Excessive preoperative fasting may paradoxically increase residual gastric volume and acidity;
- Aspiration of clear, non‐particulate gastric fluid can present as an ‘unwitnessed’ event mimicking acute peri‐induction bronchospasm or anaphylaxis;
- Point‐of‐care gastric ultrasound (POCUS) represents an invaluable tool to objectively assess gastric content and enhance preoperative risk stratification beyond mere time‐based guidelines.

## Author Contributions

**Tommaso Foggi Viligiardi:** writing – original draft, writing – review and editing, conceptualization, methodology, software, data curation, validation, formal analysis, project administration, resources, visualization, investigation, funding acquisition, supervision. **Giulia Borsotti:** supervision, resources. **Gabriele Baldini:** validation, formal analysis, writing – review and editing, supervision. **Chiara Adembri:** validation, formal analysis, writing – review and editing, supervision.

## Funding

The authors have nothing to report.

## Consent

This case report was published with the written consent of the patient.

## Conflicts of Interest

The authors declare no conflicts of interest.

## Data Availability

The data that support the findings of this study are available on request from the corresponding author. The data are not publicly available due to privacy or ethical restrictions.
